# Efficacy and feasibility of snare-assisted endoscopic resection of small submucosal gastric tumors: A retrospective analysis

**DOI:** 10.3389/fonc.2022.1068183

**Published:** 2022-11-29

**Authors:** Fei Zhao, Zhenzhen Liu, Anwei Wei, Wei Wang, Na Xu, Xuanmei Pu

**Affiliations:** Endoscopic Diagnosis and Treatment Center, The Affiliated Cancer Hospital of Zhengzhou University and Henan Cancer Hospital, Zhengzhou, China

**Keywords:** gastrointestinal tumors, submucosal gastric tumors, small gastric tumors, endoscopic resection, snare resection

## Abstract

**Background and aim:**

The prevalence of small submucosal gastric tumors is rising. Despite the fact that high success rate of endoscopic resection of small submucosal gastric tumors originating from the muscularis propria has been reported, the procedure is technically challenging and has a high rate of complications. In this study, we investigated the efficacy and feasibility of a novel snare-assisted endoscopic resection technique for small submucosal gastric tumors.

**Patients and methods:**

This is a single-center consecutive study of 50 patients who were diagnosed with small submucosal gastric tumors originating from the muscularis propria and who subsequently underwent snare-assisted endoscopic resection between January 2019 and January 2021 at our hospital. Data on the demographic characteristics, procedural success rate, complications, recurrence rate, and histopathology of the resected specimen were collected and analyzed retrospectively.

**Results:**

The majority of the patient’s population was male (66%) with the mean age of 48.4 ± 9 years (range, 20–70 years). The mean size of the tumor confirmed by endoscopic ultrasonography was 6.4 ± 1.6 mm (range, 3.1–9.8 mm). All the tumors were resected successfully using snare-assisted endoscopic resection technique. The mean procedure time was 22.8 ± 9.6 (range, 15–35 min). The technical (performed the procedure successfully without converting to surgery) and clinical (the patient fully recovered after the procedure without experiencing any complications) success rate of the procedure was 100%. Eighteen (24%) patients had very small amount of mucosal damage, and wound closure was not needed in these patients. During 6–24 months of follow-up, no recurrence or metastasis occurred. No adverse event was noted during the follow-up time.

**Conclusion:**

The novel approach of snare-assisted endoscopic resection is simple, feasible, and effective for tumors with small size and originating from the gastric muscularis propria. In addition, it offers a better alternative therapy for the complete resection of small submucosal gastric tumors. Its advantages compared with traditional endoscopic approaches such as endoscopic submucosal resection and endoscopic submucosal dissection include shorter procedure times, lesser cost, and a lower rate of complications (perforation, bleeding, and infection).

## Introduction

Gastrointestinal small submucosal tumors are prevalent and most often detected in the gastric cavity and are thought to have a high risk of malignancy (1, 2). Small gastric submucosal tumors are typically found accidentally during the routine upper gastrointestinal endoscopic examination (3). They may be asymptomatic or occasionally manifest with vague gastrointestinal symptoms. Recent improvements in endoscopic ultrasonography and the widespread use of digestive endoscopy have improved the frequency of diagnosing smaller submucosal gastric tumors, including tumors with malignant potential such as gastrointestinal stromal tumors (1, 4).

Traditionally, small submucosal gastric tumors are managed with laparoscopic surgery (5). However, with the revolution in interventional endoscopy, minimally invasive endoscopic resection techniques have become very popular for the removal of these small submucosal gastric tumors in complete and en bloc resection manner (6, 7). At present, endoscopic mucosal resection (EMR), endoscopic submucosal dissection (ESD), submucosal tunneling endoscopic resection (STER), and endoscopic full thickness resection (EFR) are the main endoscopic methods available for the resections of gastrointestinal tumors (8–10). None are completely satisfactory in that en bloc resection for relatively larger tumors cannot be consistently guaranteed by EMR technique. On the other hand, ESD offers complete en bloc resection of the relatively large tumors and a precise pathological diagnosis, but it carries a risk of perforation, bleeding, and abdominal infection. It is also associated with longer operation time and usually can only be performed by an expert endoscopist (11, 12). Small submucosal gastric tumors arising from the muscularis propria have been reported to have high (up to 50%) perforation rate following endoscopic submucosal dissection (13). If curative therapy is delayed after a perforation, the patient may probably have hemorrhagic shock or even death afterwards.

For this reason, a safe, simple, less expensive technique that could significantly lower the procedure-related complications such as gastric perforation rate is preferred. In this study, we share our experience with a cutting edge endoscopic resection technique using snare assistance for small submucosal gastric tumors. The aim of the study was to investigate the efficacy and feasibly of the snare-assisted endoscopic resection technique, and we suggest that snare-assisted endoscopic resection will provide an improved and effective alternative approach for the management of small submucosal gastric tumors.

## Material and methods

### Patients

Between January 2019 and January 2021, a total of 50 consecutive patients with small submucosal gastric tumors (<2 cm) were treated with snare-assisted endoscopic resections at our hospital. All tumors were defined by endoscopic ultrasonography (EUS) or computed tomography (CT) before the endoscopic resection. This is a retrospective analysis of consecutive patients based on the following inclusion criteria: 1) presence of small submucosal gastric tumors originating from the muscularis propria layer of the stomach without any sign of ulceration, 2) tumor diameter no more than 2 cm as measured by EUS or CT, 3) no evidence of distant metastasis, 4) no lymph node involvement, and 5) patients only managed by snare-assisted endoscopic resection. Exclusion criteria were as follows: 1) tumor diameter larger than 2 cm, 2) patients with severe bleeding or coagulation disorders, 3) patients under age of 18 or older than 75, 4) patients unable to bear general anesthesia, and 4) patients who refused to give consent for the procedure. All the procedures were performed by gastroenterologists with experience of approximately 200–300 therapeutic endoscopic procedures such as ESD.

### Definitions

The technical success rate was defined as when the procedure was successfully performed and completed without converting to surgery. The clinical success rate was defined as when the patient fully recovered after the procedure without any complications. The procedure time was defined as the time from endoscope entry into the gastric cavity to remove the lesion out. En bloc/complete resection was defined as the successful resection of the tumor in one piece.

### Procedure

**Figure 1:**
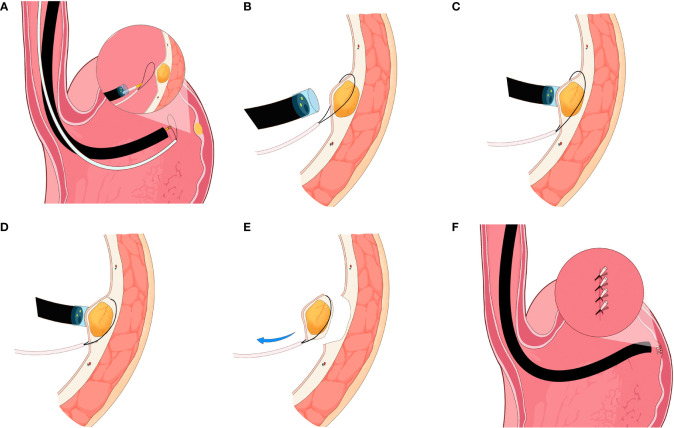
Infographics of snare-assisted endoscopic resection technique. **(A)** Endoscope attached with snare at the tip was inserted into the gastric cavity and view of the small submucosal gastric tumors. **(B)** Snare ligation around the base of the tumor. **(C)** Suction of the tumor into the transparent cap and snare tightening around the base of the tumor. **(D)** Complete resection of the small submucosal gastric tumors using snare assistance. **(E)** Resected tumor and view of the lesion resection site. **(F)** Endoscopic clipping to close the lesion resection site.

All the procedures were performed under general anesthesia in the endoscopy center of our institution. Prior to the procedure, routine bowel preparations were performed, and gastric cleansing was done using a saline solution. For the main procedure, a single-channel flexible endoscope (EVIS GIF-Q260J, Olympus, Tokyo, Japan) with transparent cap (Olympus, Model No. D-201-11804) and a snare (Boston Scientific, Ref; M00562650) attached on the tip was inserted into the gastric cavity. The lesion was examined carefully and sucked into the transparent cap. The snare was then used to ligate the base of the tumor. To resect the lesion completely, the wire of the snare was loosened to grasp the lesion from the base and then tightened. The high-frequency current was used to resect the lesion in one piece (**Figure 1**; Infographics). Hemostasis was achieved using electrocoagulation forceps (ICC 200 EA INT; ERBE, Tübingen, Germany) in case of bleeding. Finally, two to three endoclips depending on the resection size were used to close the wound site. The resected specimen was sent to the lab for histopathological diagnosis.

### Follow-up

All patients were followed up after the resection of their tumors for healing of the wound, complications, and recurrence of the tumor. During the follow-up period, patients were advised to visit the hospital for evaluation if any discomfort, such as abdominal pain or other general gastrointestinal symptoms, occurred. Endoscopic examination was performed to follow up all the patients who visited the hospital. However, for those patients who were unable to come in for follow-up visit, telephone interviews were conducted, and records of their post-procedure health including the results of their check-up at local hospitals were collected and analyzed. Forty-four (88%) patients were followed up successfully; however, we lost contact with eight (12%) patients during the follow up period.

## Results

This is a single-center analysis of 50 consecutive patients who were diagnosed with small submucosal gastric tumors and subsequently underwent snare-assisted endoscopic resection. The majority of the patient’s population was male (66%) with the mean age of 48.4 ± 9 years (range, 20–70 years). The mean size of the tumor confirmed by endoscopic ultrasound was 6.4 ± 1.6 mm (range, 3.1–9.8 mm). The mean procedure time was 22.8 ± 9.6 (range, 15–35 min). In 47 (94%) patients, the procedure was completed in <25 min. The technical (performed the procedure successfully without converting to surgery) and clinical (the patient fully recovered after the procedure without experiencing any complications) success rate of the procedure was 100%. The endoscopic clipping to close the wound was required in 64% of the patients, and 24% patients had very small amount of mucosal damage during the procedure, in which wound closure was not needed in these patients.

Tumor location of the tumors were as follows: 25 (50%) in the body of the stomach, 22 (44%) in the fundus of the stomach near the cardia, and 3 (6%) were found in the antrum. During 6–24 months of follow up, no recurrence or metastasis was noted in any of the patient. No short- or long-term complication was observed in any of the patients. The most common tumor confirmed by histopathology examination was gastrointestinal stromal tumors (62%), leiomyoma (13%), schwannoma (4%), ectopic pancreas (4%), neurofibroma (2%), and neuroendocrine tumor (2%).

## Discussion

During the routine upper gastrointestinal endoscopic examinations, a growing number of small submucosal gastric tumors, particularly those originating from the muscularis propria, are accidentally found (14). A variety of benign and malignant tumors, including gastrointestinal submucosal stromal tumors, leiomyoma, schwannoma, ectopic pancreas, neurofibroma, and neuroendocrine tumor are included in small submucosal gastric tumors (15). At present, the selection of which treatment strategy is used mainly depends on the tumor size. The American Society for Gastrointestinal Endoscopy (ASGE) guidelines in 2017 suggested endoscopic resection for tumors larger than 2 cm in size and periodic follow-up for tumors that are smaller than 2 cm in size and asymptomatic (15, 16). Contrarily, a number of recent studies suggested that small submucosal gastric tumors of smaller than 2 cm in size should also be resected endoscopically because they might be malignant.

There are several traditional endoscopic resection methods available for the resection of small submucosal gastric tumors such as EMR, ESD, and STER. However, EMR cannot perform complete en bloc resection of the lesion, and ESDs are crippled with serval complications such as perforations, bleeding, and infections (17–21). Therefore, a variety of advancements have been made in endoscopic resection techniques to reduce the complications rate and to prevent conversion rate to laparoscopic or open surgery during the procedure (22–24). In this study, we introduced a cutting edge snare-assisted endoscopic resection for small submucosal gastric tumors. The snare-assisted resection technique has several advantages over the traditional endoscopic resection techniques: (1) clear view of the dissection plane; (2) less procedure time; (3) lower cost; (4) reduced intra- and post-procedural complication rate (e.g., less bleeding); (5) shorter hospital stay duration and quick recovery after the procedure; (6) wound closure not necessary after the resection for tumors <1 cm in size; (7) stronger traction force for small submucosal gastric tumors, which is crucial for complete and smooth dissection of the tumor; and (8) endoscopic resection of small tumors using a snare assistance easy to master and perform.

As noted above, the snare-assisted resection method seems to be very crucial for the prompt removal of small submucosal gastric tumors, as the timely resection of these tumors reduces the patient’s psychological stress and eliminates non-specific gastrointestinal symptoms. Therefore, this is an appropriate option for patients with small submucosal gastric tumors who choose to have their small gastric tumors removed rather than being followed up. Our study, to best of our knowledge, is the largest to assess the efficacy and feasibility of novel snare-assisted endoscopic resection of small submucosal gastric tumors arising from the muscularis propria layer of the stomach.

Nonetheless, the snare-assisted endoscopic resection method has several limitations/disadvantages: (1) tumors located in the gastric cardia cannot be effectively resected with the assistance of a snare, as there is limited space for the lesion resection and removal; (2) tumors larger than 2 cm cannot be dissected; (3) complications cannot be completely avoided as it still carries the risk of perforation, although it is very rare; and (4) dissection of gastric small submucosal gastric tumors with a wide base may cause a wide defect in the gastric wall, which may lead to bleeding and perforations.

This study has several limitations including single-center analysis, retrospective design of the study, small sample size, and absence of control group. Further studies especially prospective clinical trials comparing snare-assisted resection with the traditional endoscopic resection are needed before the final recommendations are made on the efficacy and feasibility of snare-assisted endoscopic resection technique.

## Conclusion

Although traditional therapeutic endoscopic techniques such as EMR, ESD, STER, and EFTR are efficient and frequently used for the resection of relatively larger size tumors, an alternative method that shortens surgery time, is affordable, has fewer complications, shortens hospital stay duration, results in fast patient recovery after the procedure, and is easy to perform would be desirable. This is an effective and feasible technique that has clinical success rates that are at par with or higher than those of other endoscopic resection methods (e.g., EMR and ESD). Further prospective multi-centered randomized-controlled studies are needed.

## Data availability statement

The raw data supporting the conclusions of this article will be made available by the authors, without undue reservation.

## Ethics statement

The studies involving human participants were reviewed and approved by The Affiliated Cancer Hospital of Zhengzhou University and Henan Cancer Hospital, Zhengzhou. The patients/participants provided their written informed consent to participate in this study.

## Author contributions

Study Concept and Design: FZ, ZL. Manuscript Writing: FZ, ZL, AW, WW, NX, XP. Collection of Data and Data Analysis: AW, WW, NX, XP. Critical revision of the Manuscript: FZ, ZL. All authors contributed to the article and approved the submitted version.

## Conflict of interest

The authors declare that the research was conducted in the absence of any commercial or financial relationships that could be construed as a potential conflict of interest.

## Publisher’s note

All claims expressed in this article are solely those of the authors and do not necessarily represent those of their affiliated organizations, or those of the publisher, the editors and the reviewers. Any product that may be evaluated in this article, or claim that may be made by its manufacturer, is not guaranteed or endorsed by the publisher.

## References

[1] Nishida T Goto O Raut CP Yahagi N . Diagnostic and treatment strategy for small gastrointestinal stromal tumors. Cancer (2016) 122(20):3110–8. doi: 10.1002/cncr.30239 PMC5096017 27478963

[2] Adachi A Hirata Y Kawamura H Harada T Hattori R Kumai D . Efficacy of mucosal cutting biopsy for the histopathological diagnosis of gastric submucosal tumors. Case Rep Gastroenterol (2019) 13(1):185–94. doi: 10.1159/000499442 PMC6514511 31123445

[3] Kadkhodayan K Rafiq E Hawes RH . Endoscopic evaluation and management of gastric stromal tumors. Curr Treat Options Gastroenterol (2017) 15(4):691–700. doi: 10.1007/s11938-017-0160-0 29075963

[4] Nishida T Kawai N Yamaguchi S Nishida Y . Submucosal tumors: comprehensive guide for the diagnosis and therapy of gastrointestinal submucosal tumors. Dig Endosc. (2013) 25(5):479–89. doi: 10.1111/den.12149 23902569

[5] Conrad C Nedelcu M Ogiso S Aloia TA Vauthey JN Gayet B . Laparoscopic intragastric surgery for early gastric cancer and gastrointestinal stromal tumors. Ann Surg Oncol (2014) 21(8):2620. doi: 10.1245/s10434-013-3432-5 24647675

[6] Zhang Y Ye LP Mao XL . Endoscopic treatments for small gastric subepithelial tumors originating from muscularis propria layer. World J Gastroenterol (2015) 21(32):9503–11. doi: 10.3748/wjg.v21.i32.9503 PMC4548111 26327758

[7] Zhu L Khan S Hui Y Zhao J Li B Ma S . Treatment recommendations for small gastric gastrointestinal stromal tumors: positive endoscopic resection. Scand J Gastroenterol (2019) 54(3):297–302. doi: 10.1080/00365521.2019 30907165

[8] Nishizawa T Yahagi N . Endoscopic mucosal resection and endoscopic submucosal dissection: technique and new directions. Curr Opin Gastroenterol (2017) 33(5):315–9. doi: 10.1097/MOG.0000000000000388 28704212

[9] Schmidt A Bauder M Riecken B von Renteln D Muehleisen H Caca K . Endoscopic full-thickness resection of gastric subepithelial tumors: a single-center series. Endoscopy (2015) 47(2):154–8. doi: 10.1055/s-0034-1390786 25380509

[10] Ge N Hu JL Yang F Yang F Sun SY . Endoscopic full-thickness resection for treating small tumors originating from the muscularis propria in the gastric fundus: An improvement in technique over 15 years. World J Gastrointest Oncol (2019) 11(11):1054–64. doi: 10.4251/wjo.v11.i11.1054 PMC6883187 31798785

[11] Li P Li S Liu S Zhang D . Risk factors for complications of therapeutic endoscopy for upper gastrointestinal subepithelial lesions. Zhong Nan Da Xue Xue Bao Yi Xue Ban. English, Chinese (2021) 46(3):278–82. doi: 10.11817/j.issn.1672-7347.2021 PMC10929934 33927075

[12] Natsagdorj E Kim SG Choi J Kang S Kim B Lee E . Clinical outcomes of endoscopic submucosal dissection for early gastric cancer in patients with comorbidities. J Gastric Cancer. (2021) 21(3):258–67. doi: 10.5230/jgc.2021.21.e25 PMC8505124 34691810

[13] Li S Liang X Zhang B Tao X Deng L . Novel endoscopic management for small gastric submucosal tumors: A single-center experience (with video). Dig Liver Dis (2021) 53(7):895–9. doi: 10.1016/j.dld.2021.02.014 33737005

[14] Pimentel-Nunes P Libânio D Bastiaansen BAJ Bhandari P Bisschops R Bourke MJ . Endoscopic submucosal dissection for superficial gastrointestinal lesions: European society of gastrointestinal endoscopy (ESGE) guideline - update 2022. Endoscopy (2022) 54(6):591–622. doi: 10.1055/a-1811-7025 35523224

[15] Esaki M Ihara E Gotoda T . Endoscopic instruments and techniques in endoscopic submucosal dissection for early gastric cancer. Expert Rev Gastroenterol Hepatol (2021) 15(9):1009–20. doi: 10.1080/17474124.2021.1924056 33909540

[16] Akahoshi K Oya M Koga T Shiratsuchi Y . Current clinical management of gastrointestinal stromal tumor. World J Gastroenterol (2018) 24(26):2806–17. doi: 10.3748/wjg.v24.i26.2806 PMC6048423 30018476

[17] Kahoshi K Oya M Koga T Koga H Motomura Y Kubokawa M . Clinical usefulness of endoscopic ultrasound-guided fine needle aspiration for gastric subepithelial lesions smaller than 2 cm. J Gastrointestin Liver Dis (2014) 23(4):405–12. doi: 10.15403/jgld.2014.1121.234.eug 25531999

[18] Ye LP Zhang Y Mao XL Zhu LH Zhou X Chen JY . Submucosal tunneling endoscopic resection for small upper gastrointestinal subepithelial tumors originating from the muscularis propria layer. Surg Endosc. (2014) 28(2):524–30. doi: 10.1007/s00464-013-3197-8 24013472

[19] Cai MY Zhu BQ Xu MD Qin WZ Zhang YQ Chen WF . Submucosal tunnel endoscopic resection for extraluminal tumors: a novel endoscopic method for en bloc resection of predominant extraluminal growing subepithelial tumors or extra-gastrointestinal tumors (with videos). Gastrointest Endosc. (2018) 88(1):160–7. doi: 10.1016/j.gie.2018.02.032 29499127

[20] Cai MY Martin Carreras-Presas F Zhou PH . Endoscopic full-thickness resection for gastrointestinal submucosal tumors. Dig Endosc. (2018) Suppl 1:17–24. doi: 10.1111/den.13003 29658639

[21] Li B Chen T Qi ZP Yao LQ Xu MD Shi Q . Efficacy and safety of endoscopic resection for small submucosal tumors originating from the muscularis propria layer in the gastric fundus. Surg Endosc. (2019) 33(8):2553–61. doi: 10.1007/s00464-018-6549-6 30478693

[22] Zhang Q Cai JQ Wang Z . Usefulness of tumor traction with a snare and endoclips in gastric submucosal tumor resection: a propensity-score-matching analysis. Gastroenterol Rep (Oxf). (2020) 9(2):125–32. doi: 10.1093/gastro/goaa050 PMC8128025 34026219

[23] Zhang Q Lian ZY Cai JQ Bai Y Wang Z . Safety and effectiveness of mucosal traction using a snare combined with endoclips to assist the resection of esophageal intraepithelial neoplasia: a propensity score matching analysis. Dis Esophagus (2022) 35(1):doab018. doi: 10.1093/dote/doab018 33870425

[24] Zhang Q Cai JQ Wang Z Xiao B Bai Y . Snare combined with endoscopic clips in endoscopic resection of gastric submucosal tumor: a method of tumor traction. Endosc Int Open (2019) 7(9):E1150–62. doi: 10.1055/a-0849-9625 PMC6715454 31475234

